# *Agave amica* (Medik.) Thiede & Govaerts (Asparagaceae)—Insights into Its Valuable Phenolic Profile and *In Vitro* Antimicrobial, Antibiofilm, Antioxidative, and Antiproliferative Properties

**DOI:** 10.3390/antibiotics14070638

**Published:** 2025-06-23

**Authors:** Mihaela Niculae, Daniela Hanganu, Ilioara Oniga, Sergiu-Alexandru Burcă, Brîndușa Tiperciuc, Irina Ielciu, Emoke Pall, Timea Bab, Ramona Flavia Burtescu, Mihaela Andreea Sava, Daniela Benedec

**Affiliations:** 1 Department of Clinical Sciences, University of Agricultural Sciences and Veterinary Medicine Cluj-Napoca, 400372 Cluj-Napoca, Romania; mihaela.niculae@usamvcluj.ro (M.N.); emoke.pall@usamvcluj.ro (E.P.); 2 Department of Pharmacognosy, Faculty of Pharmacy, “Iuliu Hattieganu” University of Medicine and Pharmacy, 400010 Cluj-Napoca, Romania; ioniga@umfcluj.ro (I.O.); burca.sergiu.alex@elearn.umfcluj.ro (S.-A.B.); bab.timea.henrietta@elearn.umfcluj.ro (T.B.); dbenedec@umfcluj.ro (D.B.); 3 Department of Pharmaceutical Chemistry, “Iuliu Hațieganu” University of Medicine and Pharmacy, 41 V. Babeş Street, 400012 Cluj-Napoca, Romania; btiperciuc@umfcluj.ro; 4 Department of Pharmaceutical Botany, Faculty of Pharmacy, “Iuliu Haţieganu” University of Medicine and Pharmacy, 400337 Cluj-Napoca, Romania; irina.ielciu@umfcluj.ro; 5 PlantExtrakt Ltd., 407059 Cluj-Napoca, Romania; ramona.burtescu@plantextrakt.ro; 6 Transylvania School of Botanic Art & Illustration, Copșa Mare, 557046 Sibiu, Romania; mihaelaandreea.sava@yahoo.com

**Keywords:** *A. amica*, phenols, antibacterial, antifungal, antibiofilm, antioxidant, cytotoxic

## Abstract

**Background/Objectives**: *Agave amica* (Medik.) Thiede & Govaerts (Asparagaceae family) is an ornamental bulbous species, widely used for its fragrance but less studied as a medicinal species. This study is aimed at assessing the phenolic profile and selected biological properties of ethanolic extracts obtained from the aerial parts and bulbs of *A. amica* cultivated in Romania. **Methods**: The phenolic composition was characterized by spectrophotometric methods and LC/MS analysis. The antioxidant activity was evaluated by DPPH (2,2-diphenyl-1-picrylhydrazyl radical scavenging capacity) and FRAP (Ferric reducing antioxidant power) tests, while the *in vitro* antimicrobial capacity was investigated by the agar-well diffusion, the broth microdilution, and the antibiofilm assays. Cytotoxicity was tested on a colorectal adenocarcinoma cell line (DLD-1) by a CCK-8 assay. **Results**: Both ethanolic extracts showed important polyphenol content and caffeic acid as their main compound. Significantly higher amounts of total polyphenols (44.25 ± 1.08 mg/g), tannins (12.55 ± 0.34 mg/g), flavonoids (9.20 ± 0.19 mg/g), caffeic acid derivatives (19.95 ± 0.05 mg/g), and also antioxidant activity (IC_50_ = 0.82 ± 0.02 mg/mL, and 79.75 ± 1.80 µM TE/g, respectively) were found for the aerial parts extract compared to the bulbs one (*p* < 0.001). Notable anti-*Candida albicans* activity and moderate efficacy against *Listeria monocytogenes* and *Staphylococcus aureus* were displayed by both extracts against planktonic cells and biofilm. A dose-dependent cytotoxicity towards the colorectal adenocarcinoma cell line was recorded as well. **Conclusions**: This study brings novelty to the scientific literature by characterizing the phenolic profile and *in vitro* antimicrobial, antibiofilm, antioxidant, and antiproliferative activities of the ethanolic extracts obtained from *A. amica*, thus highlighting this herbal species’s medicinal potential.

## 1. Introduction

According to a 2022 World Health Organization (WHO) report, resistance to antimicrobial agents remains a major threat to global health due to an increasing number of microbial infections that are refractory to antimicrobial therapy, together with the rapid spread of multidrug-resistant strains at different community levels and settings. Several mechanisms entitle organisms to manifest resistance against conventional antibiotics as well as towards the host’s innate immune responses [1]. Among them, biofilms are an established hallmark of microbial adaptation, persistence, and virulence [1,2]. Furthermore, biofilm-forming ability was demonstrated for numerous pathogenic and opportunistic organisms [2]. To tackle this worrying and complex phenomenon, a multifaceted approach that includes targeted strategies aiming to reduce antimicrobial usage and implement surveillance and monitoring systems, as well as promoting the search and discovery of alternative therapies, is recommended [3]. In fact, developing new antimicrobial drugs and alternative therapies has crucial importance, with plant-derived extracts suggested by numerous studies as a potential source of antimicrobial bioactive components that can address several limitations and disadvantages of conventional antibiotics [4]. Certain bioactive metabolites produced by plants have shown not only antimicrobial efficacy but also antibiofilm activity against different bacterial strains [5].

*Agave amica* (Medik.) Thiede & Govaerts, tuberose, is a member of the Asparagaceae family, an ornamental bulbous species especially known for its pleasant fragrance and use in the perfumery and cosmetic industry [6,7,8,9,10]. Formerly known as *Polianthes tuberosa* L., based on recent phylogenetic studies and molecular data, it has been removed from the Agavaceae and Amaryllidaceae families systematically and included in the family Asparagaceae and subfamily Agavoideae [8,9,10,11]. This species is the only taxon of the former genus *Polianthes*, cultivated across the globe and used for medicinal, ornamental, and ceremonial practices since ancient times [11,12]. It is a perennial monocotiledonate species, native to Mexico and cultivated in China, Egypt, South Africa, India, and Japan for its fragrant flowers and the essential oil it yields [6,13,14,15]. The bulbs of the species produce flowers after four years of growth, only once, in late summer, when spikes develop from them [16]. The flowers are white, night-blooming, with single or double fragrant tepals, and are funnel-shaped while the leaves are sessile, elongated, arched, and ribbon-shaped [16,17,18]. Nowadays, it is cultivated widely in tropical, subtropical, and subtemperate areas for the fragrance of its flowers, used as flower decorations, but especially for their fragrance, with tuberose fragrant oils being one of the most highly demanded perfumes globally [15,19]. Tuberose flowers are completely edible and can be used as ingredients in different ways [16,17]. Traditionally, it is described not only as an ornamental species but also as a medicinal plant with flowers and bulbs used for its diuretic, emetic, anti-gonorrhea, anti-insomnia, antispasmodic, and anti-rashes effects [13,20]. The scientific literature suggests that this species is a promising source of bioactive compounds, such as tuberolactone and three flavonoids (kaempferol, kaempferol-3-O-xyloside, and kaempferol-3-4′-O-dixyloside), polysaccharides, polianthosides B-G, spirostanols, furostanols, steroidal glycosides, and saponins [14,21,22,23]. Volatile metabolites belong to the classes of terpenoids, benzenoids, phenylpropanoids, and fatty acid derivatives [24]. All these bioactive metabolites were linked to anti-inflammatory, antioxidant, antimicrobial, cytotoxic, membrane stabilizing, and thrombolytic activities [14,19,25,26].

Previously performed studies on this species aimed to evaluate the antibacterial, antitumor, and anti-inflammatory activities of its flower extracts including in silver, gold, and zinc nanoparticles [20,27,28,29]. The flower extracts also exhibited antimicrobial activity [21,30]. There are few other studies focusing on other parts of the species, such as the bulbs, attributing the antioxidant and antimicrobial activity to alkaloids [13] or phenols [18], or the leaves, assessing their antioxidant or antibacterial capacity attributed to polyphenols [14,31]. Only one study aims to compare the antibacterial activity of flowers, stems, and bulbs [32]. At the same time, some studies focus on the volatile metabolites of the species, studying their flower extracts [6,17,33].

In Romania, the *A. amica* species is cultivated and perfectly adapted to the pedoclimatic conditions in the whole country but research on it is very limited [34,35,36]. Taking all these factors into consideration, it appears there is currently little information on the primary and secondary metabolites of *A. amica*, and evidence of its biological activities is also reduced [16,17]. To the best of our knowledge, no previous studies on the *A. amica* polyphenolic profile and the antioxidant, antimicrobial, antibiofilm, and cytotoxic/antiproliferative properties of ethanolic extracts have been performed in Romania. Moreover, no previously performed study has aimed to study the comparison between extracts obtained from the aerial parts (Pta) and bulbs (Ptb). In this context, the present study’s main purpose is to bring novelty to the scientific literature by characterizing the ethanolic extracts obtained from *A. amica* cultured in Romania, with a special focus on the phenolic profile and selected activities, namely antibacterial, antifungal, antibiofilm, antioxidant, and cytotoxic, aiming therefore to provide scientific arguments for the medicinal uses of the species.

## 2. Results

### 2.1. Quantitative Evaluation of Phenolic Compounds and Antioxidant Activity

The contents of total polyphenols (TPC) expressed in mg GAE/g, flavonoids (FC) expressed in mg RE/g, and caffeic acid derivatives (CADC) expressed in mg CAE/g, as well as the results obtained for the evaluation of the antioxidant activity determined by DPPH and FRAP methods of the two *A. amica* extracts are presented in Table 1. It was found that the extract obtained from the aerial parts of *A. amica* (Pta) contained a significantly higher amount (*p* < 0.001) of active polyphenolic principles: total polyphenols (44.25 ± 1.08 mg/g), tannins (12.55 ± 0.34 mg/g), flavonoids (9.20 ± 0.19 mg/g), and caffeic acid derivatives (19.95 ± 0.05 mg/g) compared to the bulb extract (TPC: 29.60 ± 0.89 mg/g, TC: 9.85 ± 0.15 mg/g, FC: 5.65 ± 0.13 mg/g, and CADC: 8.08 ± 0.12 mg/g, respectively). Comparative analysis of these quantitative results showed highly statistically significant differences (*p* < 0.001) between the two *A. amica* samples (*p* < 0.001).

The results of the antioxidant activity assays performed on the *A. amica* extracts obtained from aerial parts and bulbs showed that the Pta extract presented higher antioxidant activity (IC_50_ = 0.82 ± 0.02 mg/mL, and 79.75 ± 1.80 µM TE/g, respectively) than the Ptb extract (IC_50_ = 1.65 ± 0.01 mg/mL, and 29.62 ± 0.37 µM TE/g, respectively) by both methods used. Statistical analysis of the results from the two antioxidant activity assays showed very significant differences (*p* < 0.001) between the *A. amica* extracts (*p* < 0.001), and in the case of the DPPH test, the results were less effective than the Trolox standard (*p* < 0.001).

### 2.2. LC–MS Analysis

The results obtained using LC–MS identification and quantification of the individual components of the two *A. amica* samples can be found in Table 2 and Supplementary Materials Figures S1–S16.

The compositions of these samples highlight that caffeic acid was the main compound, with its highest amount being found in the Pta samples (8890 ± 22.2 μg/g). This compound is accompanied by other phenolic acids important for biological effects, namely chlorogenic acid and salicylic acid (present only in the Pta sample). Flavonoids are represented by both glycosylated and free structures (aglycons), such as hyperoside, luteolin-7-*O*-glucoside, chrysin, luteolin, naringenin, and quercetin. For the Pta sample, luteolin was the flavonoidic compound found with the highest amount (2300 ± 5.7 μg/g), followed by luteolin-7-*O*-glucoside (2290 ± 5.7 μg/g), chrysin (1920 ± 4.7 μg/g), hyperoside (1640 ± 4.1 μg/g), naringenin (45 ± 0.2 μg/g), and quercetin (20 ± 0.09 μg/g). For the Ptb sample, only glycosylated structures, namely hyperoside and luteolin-7-*O*-glucosid, in important amounts (1510 ± 3.7 μg/g and, respectively, 72 ± 0.4 μg/g) were found. The LC–MS analysis also confirms the lower polyphenol content in the Ptb sample compared to the Pta sample.

### 2.3. Antiproliferative Assays

**Figure 1:**
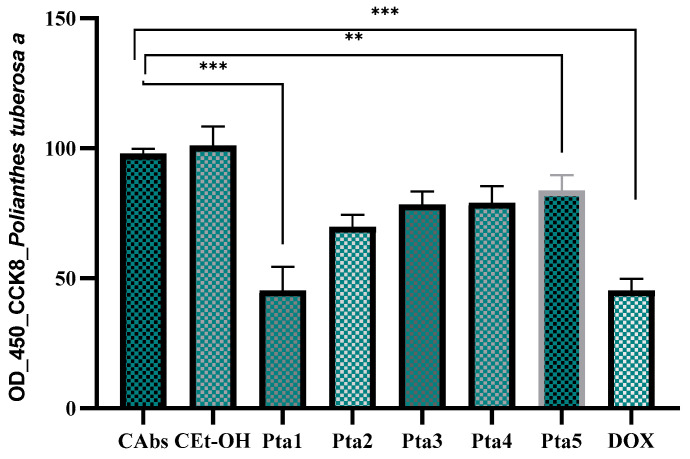
The effect of *A. amica* aerial parts extract on the growth inhibition of DLD cells was evaluated by incubating the cells with different concentrations of the extract (Pta5—0.130 μmol GAE/μL, Pta4—0.260 μmol GAE/μL, Pta3—0.390 μmol GAE/μL, Pta2—0.520 μmol GAE/μL, Pta1—0.650 μmol GAE/μL) for 24 h. Cell proliferation was measured using the CCK-8 assay. Doxorubicin was utilized as a positive control, and statistical significance was determined with ** *p* ≤ 0.01, and *** *p* ≤ 0.001. The IC_50_ value through dose-response analysis revealed an inhibitory concentration of 0.580 μmol GAE/μL.

The antiproliferative effects of *A. amica* extracts (Pta and Ptb) were evaluated by measuring the absorbance of colorectal adenocarcinoma cells (DLD-1) after treatment. The CAbs (absolute control) group exhibited the highest absorbance, indicating minimal interference with cell viability. In contrast, the cultures treated with ethanol showed only a marginal reduction in absorbance, suggesting that ethanol did not significantly affect cell viability relative to the baseline control. Treatment with the highest concentration of the Pta extract resulted in a dose-dependent decrease in absorbance, with Pta5 demonstrating the most pronounced effect, as evidenced by the lowest absorbance values. The DOX (doxorubicin) group, serving as the positive control, consistently exhibited the lowest absorbance values, reflecting its potent cytotoxicity. The observed reductions in absorbance for the Pta groups, particularly for Pta5, indicate a notable antiproliferative effect; however, the efficacy of Pta extracts remained weaker compared to doxorubicin (Figure 1).

**Figure 2:**
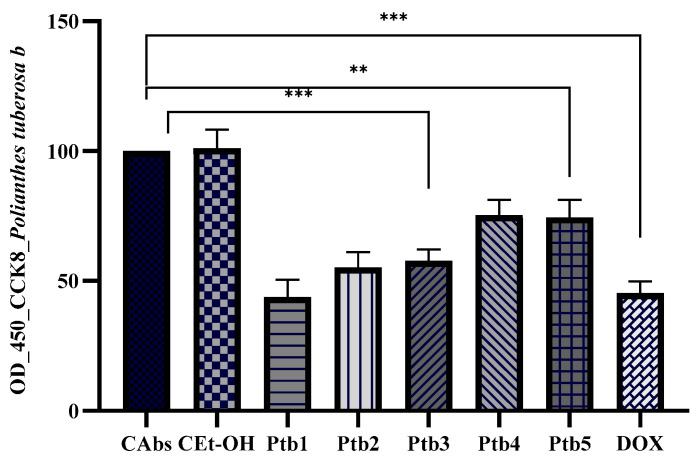
The effect of *A. amica* b extract on the growth inhibition of DLD cells was evaluated by incubating the cells with different concentrations of the extract (Ptb5—0.087 μmol GAE/μL, Ptb4—0.174 μmol GAE/μL, Ptb3—0.261 μmol GAE/μL, Ptb2—0.348 μmol GAE/μL, Ptb1—0.435 μmol GAE/μL) for 24 h. Cell proliferation was measured using the CCK-8 assay. Doxorubicin was utilized as a positive control, and statistical significance was determined with ** *p* ≤ 0.01, and *** *p* ≤ 0.001. The IC_50_ value through dose-response analysis revealed an inhibitory concentration of 0.285 μmol GAE/μL.

The potential antiproliferative activity of Ptb (assessed at five different concentrations, between 0.087 and 0.435 μmol GAE/μL) was evaluated by measuring the absorbance of the chromogenic reaction following the CCK-8 assay. Our results indicate a dose-dependent cytotoxic effect of the Ptb extract, as evidenced by the progressive decrease in absorbance values with increasing extract concentration (Figure 2). The highest concentration (Ptb1) exhibited the most pronounced reduction in absorbance, suggesting significant cytotoxicity and reduced cell viability. Conversely, the lowest concentration (Ptb5) displayed a comparatively higher absorbance, indicating a weaker cytotoxic effect, though still lower than the negative control (CAbs). The ethanol control (CEtOH) showed absorbance values similar to the negative control, suggesting that ethanol alone does not significantly impact cell viability. The positive control (DOX) demonstrated the lowest absorbance values, confirming its expected strong cytotoxic activity. These findings suggest that the extract exerts a dose-dependent inhibitory effect on cell viability, with higher concentrations eliciting greater cytotoxicity.

### 2.4. Antimicrobial Activity Assays

#### 2.4.1. The Agar-Well Diffusion Method

The *A. amica* extracts’ (Pta and Ptb) *in vitro* antimicrobial activity was initially investigated by a screening test, namely the agar-well diffusion method. The obtained results are expressed as diameters of the inhibition zone and are displayed in Table 3.

Both *A. amica* extracts (Pta and Ptb) were able to inhibit several tested bacterial strains; still, the *in vitro* antimicrobial potential was found to be significantly lower compared to the positive control gentamicin (*p* < 0.05 for both extracts vs. gentamicin when tested against both Gram-positive and Gram-negative strains). Furthermore, no inhibitory effect was noticed against *Enterococcus faecalis* and *Pseudomonas aeruginosa* (Table 3). Nevertheless, an interesting *in vitro* antifungal activity was indicated when comparing the obtained inhibition zone diameters with fluconazole (*p* > 0.05 for both extracts vs. fluconazole). This notable exception was further confirmed by the results of the broth microdilution method (Table 4) and the antibiofilm assay (Table 5).

#### 2.4.2. The Broth Microdilution Method

The values obtained when performing the broth microdilution method are displayed in Table 4. As indicated by the preliminary screening results, both *A. amica* extracts were able to exhibit a certain degree of *in vitro* antimicrobial efficacy manifested as both inhibitory (MIC) and antimicrobicidal (MBC) activity against the tested organisms. The established values of MIC and MBC generally involved either the lowest tested dilution (against MRSA, *Bacillus cereus*, and *Escherichia coli*) or the second most concentrated form of the extracts (against *Listeria monocytogenes* and MSSA). While such high concentrations of the tested products were required against bacterial strains, *Candida albicans* efficacy of both extracts was further underlined by the four-fold lower MIC and MFC values.

#### 2.4.3. The Antibiofilm Assay

Given the results of the previous two assays, the two *A. amica* extracts’ antibiofilm properties were further investigated towards three organisms: two Gram-positive bacteria (MSSA and *Listeria monocytogenes*) and a fungus (*Candida albicans*), with the results presented in Table 5. Using a previously described protocol, inhibition percentage values (%) were calculated and interpreted as good (above 50%, ++), poor (0–50%, +), and no inhibition or enhancement of biofilm development and growth (<0) [37]. Thus, considering these calculated values (%), good antibiofilm activity (above 50%, ++) was observed for both extracts when tested against all organisms for the T24 stage (destruction of 24 h pre-formed biofilm). Regardless of the extract and bacterial strain, the effect on the T0 stage (biofilm attachment) was recorded as rather poor (0–50%, +) and no inhibition or enhancement of biofilm development and growth (<0) (Table 5).

## 3. Discussion

*A. amica* is a species cultivated mainly for its ornamental properties, but it is also endowed with medicinal properties. The extracts from its bulbs, flowers, and aerial parts are traditionally used in some countries (in Bangladesh, Algeria, India, and Mexico) for their diuretic, laxative, emetic, uterine relaxant, and antigonorrhoeic properties, and the healing of pediatric rashes, and scientific studies have shown antifungal, antibacterial, anti-MRSA, nematicidal, pesticidal, mosquito larvicidal antioxidant, and antitumor actions in the liver, pancreatic cancers, and promyelocytic leukemia [13,22,23,24,31,32,38]. In Romania, tuberose is widely grown as an ornamental plant, but pharmacognostic and pharmacological research regarding it is lacking or very limited.

This research brings novelty by the comparative testing, for the first time in Romania and probably in Europe, of the polyphenolic profile and the antioxidant, antimicrobial, and especially antibiofilm and cytotoxic/antiproliferative properties of ethanolic extracts (70% ethanol in water *v*/*v*) obtained from the aerial parts (Pta) and bulbs (Ptb) of *A. amica*. Following spectrophotometric evaluations, it was found that the concentration of polyphenolic active principles is different, and the Pta extract is richer in phenolic compounds (TPC: 44.25 mg/g, TC: 12.55 mg/g, TF: 9.20 mg/g, and CADC: 19.95 mg/g, respectively) than Ptb (TPC: 29.60 mg/g, TC: 9.85 mg/g, FC: 5.65 mg/g, and CADC: 8.08 mg/g, respectively). The amounts of TP, flavonoids, and tannins in the Pta sample proved to be higher compared to a crude methanolic extract obtained from *A. amica* leaves from Algeria [13]. Regarding the presence of caffeic acid derivatives (CADC), the Pta sample presented a higher CADC content (19.95 mg/g) than the Ptb sample (8.08 mg/g), with there being no other specialized published works in this regard.

The main constituents of the volatile components in *A. amica* flower extracts are represented by benzyl benzoate, methyl 2-amino benzoate, methyl isoeugenol, isoeugenol, benzyl salicylate, methyl salicylate, geraniol, and 1,8-cineole. As for the total phenolic content of floral extracts, variations are reported in relation to the type of solvent (0.094, 0.18, 0.14, 0.007, 0.004, and 0.110 mg gallic acid equivalent/mg fresh weight in water, methanol, ethanol, ethyl acetate, hexane, and dichloromethane extracts, respectively) [21].

The antioxidant potential of *A. amica* flower extracts was previously reported [18,21]. The highest values were noticed for the methanol, dichloromethane, and aqueous soluble fractions by DPPH and ABTS assays, while the best results of the FRAP assay were obtained for the aqueous extract [21]. The phenolic content of 113.49 mg of GAE/g of extractives and a significant free radical scavenging activity with IC50 values of 71.23 and 84.27 µg/mL, respectively, characterized a crude methanol extract and its hexane soluble fraction [18].

In our study, the antioxidant activity was evaluated by the *in vitro* DPPH and FRAP methods, demonstrating an important antioxidant potential of the extracts obtained from the aerial parts and bulbs of *A. amica* cultivated in Romania. The aerial parts showed a higher DPPH radical scavenging activity than the bulbs, with the effect being possibly due to higher concentrations of caffeic acid (8890 μg/g) and chlorogenic acid (4000 μg/g) in the Pta extract compared to Ptb (4230 μg/g caffeic acid and 1501 μg/g chlorogenic acid). Furthermore, the electron-donating capacity of the *A. amica* extracts was measured by the FRAP method. The capacity to reduce Ferric ions (Fe^3+^) to ferrous ions (Fe^2+^) was higher for the aerial parts (79.75 ± 1.80 µM TE/g) than for the bulbs (29.62 ± 0.37 µM TE/g). Therefore, the positive correlation between antioxidant activity and total polyphenolic compound content (*p* < 0.001) suggests that these secondary active principles act as reducing agents and hydrogen donors, and may exert an important antioxidant capacity, as also reported in other publications [13,18].

The cytotoxicity assay results indicate that both extracts (Pta and Ptb) exhibit dose-dependent inhibitory effects on the human colorectal adenocarcinoma cell line DLD-1 cell viability, with higher concentrations leading to increased cytotoxicity. While Ptb shows slightly enhanced cytotoxicity compared to Pta, this difference is relatively modest, particularly at lower concentrations. The absorbance values for Ptb1 are notably lower than those for Pta1, suggesting a stronger inhibitory effect at the highest concentration. However, at the lowest tested concentration (Ptb5 vs. Pta5), the difference in viability reduction is only approximately 10%, indicating that the two extracts have comparable effects at lower doses. The CEtOH exhibits similar values to the negative control, confirming that ethanol itself does not influence cell viability. As expected, the positive control demonstrates the most significant cytotoxic activity. These findings suggest that while Ptb may have a slightly higher potency than Pta, the overall differences are not substantial, particularly at lower concentrations. Further investigations, including chemical composition analysis and mechanistic studies, are necessary to determine whether Ptb possesses a higher concentration of bioactive compounds or if other factors contribute to its slightly stronger cytotoxic effect. Cytotoxicity against tumoral cells was previously reported only by a limited number of studies [20,27,28]. In fact, gold nanoparticles obtained from the floral aqueous extract of *A. amica* (PtubAuNPs) manifested *in vitro* toxicity towards the MCF 7 breast cancer cell line at a concentration of 100 µg/mL and induced these cells’ apoptosis in a dose-dependent manner [28]. Furthermore, silver nanoparticle products (PTAgNPs) obtained from *A. amica* aqueous root extract at concentrations of 5, 10, 15, 20, 25, 30, 35, 40, 45, and 50 µg/mL produced Vero cell line morphologic alterations such as cell shrinkage and the loss of colony formation ability [20]. The cytotoxicity of PTAgNPs was demonstrated also on the A-431 melanoma cell line based on the results of a complex protocol that included the MTT assay, cell cycle analysis, the Comet assay, and reactive oxygen species (ROS) level determination: dose-dependent toxicity with an IC_50_ of 56.54 µg/mL, S phase arrest, as well as A431 cell death by inducing ROS [27]. Moreover, using the brine shrimp lethality bioassay, Rumi et al. reported strong cytotoxic activity with an LC_50_ value of 3.56 and 9.31 μg/mL, respectively, for a crude methanol extract and its carbon tetrachloride soluble fraction compared to standard vincristine sulfate (VS) [18]. According to the literature data, polyphenolic compounds present in high concentrations in analyzed extracts such as salicylic acid, chlorogenic acid, caffeic acid, chrysin, luteolin, and hyperoside have shown promising potential in the prevention and treatment of colorectal cancer, through multiple biological mechanisms. They act by inhibiting cell proliferation, inducing apoptosis, blocking cyclin-dependent kinases, reducing inflammation, and modulating signaling pathways involved in carcinogenesis such as Wnt/PI3K/AKT, NF-κB, and PD-L1 [39,40,41,42,43]. Among them, hyperoside stands out for its solid preclinical results in lung cancer, as well as its potential applicability in colorectal cancer [40]. Also, metabolites of salicylic acid have shown notable inhibitory effects on tumor cell division [7]. Overall, these natural compounds may constitute valuable adjuvants in oncological therapies, but rigorous clinical studies are needed to validate their efficacy and safety. Also, optimizing bioavailability and identifying effective doses remain essential challenges for their therapeutic implementation. The presence of these compounds in extracts along with other polyphenols was an important premise in testing the extracts on DLD-1 cell lines and also supported the explanation of antitumor mechanisms.

The testing protocol employed in our study indicated notable anti-*Candida albicans* activity as well as moderate efficacy against *Listeria monocytogenes*, and *Staphylococcus aureus* was displayed by both extracts against planktonic cells and biofilms. In relation to the antimicrobial potential, our results are in agreement with those reported by Rumi et al. Using the disk diffusion test, these authors found mild to moderate activity (zone of inhibition = 9.0–15.0 mm) against *Bacilllus megaterium*, *B. cereus*, *Sarcina lutea, Salmonella paratyphi*, and *Vibrio mimicus* for a crude methanol extract obtained from *A. amica* tubers originating from Bangladesh [18]. More recently, *Staphylococcus aureus* efficacy was reported for crude extracts obtained from the leaves of *A. amica* cultivated in Bangladesh, with inhibition zone diameters ranging from 7 to 21 mm, and the ethyl acetate crude extract demonstrated the highest antagonism against MRSA with a minimum inhibitory concentration value of 6.25 µg/mL and a zone of inhibition of 21 mm [31].

The disk diffusion method proved for tuberose flower extracts obtained by green technology, namely supercritical carbon dioxide (SC-CO_2_) extraction and solvent extractions (using *n*-hexane and petroleum ether of b.p. 60–80 °C), *in vitro* efficacy against *Staphylococcus aureus*, *Pseudomonas aeruginosa*, *Escherichia coli*, and *Candida albicans* and no activity against *H. pylori* and *V. cholerae*. Still, the authors described significant differences when comparing the MIC values obtained for the two extracts against different bacterial strains, namely the *n*-hexane extract had the highest activity against *P. aeruginosa*, while the petroleum ether extract was the most active against *C. albicans*, but with no anti-*Staphylococcus aureus* potency. Moreover, these two extracts’ GC/MS analysis indicated the presence of *p*-xylene, limonene, methyl eugenol, and salicyl hydrazide, all active compounds well known for their antimicrobial potential [30]. The flower, stem, and tuber of *A. amica*-derived extracts prepared by maceration with different polarity solvents (*n*-hexane, ethyl acetate, and methanol) were reported active against *Staphylococcus epidermidis* and *Propionibacterium acnes* [32]. *A. amica* leaves’ crude ethanolic extract at concentrations of 100, 250, and 500 μg/mL (disk diffusion) inhibited the growth of *Bacillus subtilis*, *Staphylococcus aureus*, *Micrococcus luteus*, *Escherichia coli*, *Pseudomonas aeruginosa*, and *Salmonella typhi*, with the Gram-positive bacteria more susceptible compared to the Gram-negative ones. This extract was also found with significant antifungal activity against *Aspergillus niger* and *Candida albicans* [38].

Methanolic extracts prepared from the bulbs of cultivated *P. tuberosa* “Double” and six wild species: *P. platyphylla*, *P. montana*, *P. clivicola*, *P. howardii*, *P. pringlei*, and the genera *Manfreda* and *Prochnyanthes* from Mexico were able to inhibit two phytopathogens; the *Prochnyanthes* extract displayed the greatest anti-*P. aeruginosa* activity, with a percent of inhibition (PI) of 24 ± 2.34%, while the most effective against *D. dadantii* were *P. ringlei* (PI = 21.8 ± 2.39%), *Prochnyanthes* (PI = 15.5 ± 0.86%), *Manfreda* sp. (PI = 14.4 ± 1.7%), and *P. montana* (PI = 9 ± 1.16%) [44].

Previous studies reported data on the *in vitro* antifungal activity of the absolute of tuberose and its constituents against *Aspergillus niger* and *Candida albicans* [38] and *Colletotrichum gloeosporioides* [33]. The mycelial growth on a potato–dextrose–agar medium was only slightly inhibited by the tuberose absolute at a concentration of 500 mg/L, while significant activity was recorded for three constituents present in the absolute: geraniol, indole, and methyl anthranilate [33].

However, assessed by the disk diffusion method, the bulbs and bulbils alkaloid extracts obtained from *A. amica* cultivated in Algeria did not show *in vitro* inhibitory effects at a concentration of 50 mg/mL against several reference strains *(Eschericha coli* ATCC 25922, *Pseudomonas aeruginosa* ATCC 27853, *Staphylococcus aureus* ATCC 25923, *Enterococcus faecalis* ATCC 29212, *Staphylococcus aureus* resistant to Methicillin (MRSA), and *Candida albicans* ATCC 90028). Furthermore, the authors found only moderate antioxidant activity with the bulbs and bulbils alkaloid extracts exhibiting an antiradical effect with IC_50_ = 0.231 ± 0.017 mg/mL and 0.233 ± 0.093 mg/mL, respectively, compared to vitamin C with IC_50_ = 0.0194 ± 0.0002 mg/mL [13].

Based on the most recent studies on *A. amica*, green synthesized zinc oxide [29], silver [20,27], and gold [28] nanoparticle flower extract biosynthesis was associated with interesting antimicrobial and antiproliferative properties. Green synthesized *A. amica* flower concentrate zinc oxide nanoparticles (ZnONPs) inhibited the growth of selected bacterial and fungal pathogens and suppressed biofilm formation in *Pseudomonas aeruginosa* in a dose-dependent manner [29]. Gold nanoparticles obtained from the floral aqueous extract of *A. amica* (PtubAuNPs) with concentrations of 25 µg/mL, 50 µg/mL, and 75 µg/mL were able to inhibit *in vitro* the *E. coli* and *S. aureus* growth. Higher values of inhibition zone were recorded in the case of the Gram-negative strain due to the nanoparticles’ increased ability to penetrate this specific type of bacterial wall. The silver nanoparticles’ products (PTAgNPs) are reported to display a relatively more intense and broader *in vitro* antimicrobial efficacy compared to the gold ones [20,27]. Reference strains of *Staphylococcus aureus*, *Bacillus subtilis*, *Escherichia coli, Pseudomonas aeruginosa*, and *Klebsiella pneumoniae* were susceptible towards silver nanoparticles obtained from *A. amica* aqueous root extract.

Major compounds identified in the chemical composition of *A. amica* extracts evaluated in the present study, namely chlorogenic acid, caffeic acid, and salicylic acid, were previously reported with antimicrobial and antibiofilm properties through several mechanisms, including disrupting bacterial cell membranes, potentially affecting quorum sensing systems [45]. Caffeic acid’s antimicrobial and antibiofilm activity is linked to its ability to act as a cell permeabilizer, causing membrane alterations and potassium leakage. It can also affect phenylalanine ammonia-lyase (PAL) and peroxidase (POD) activity, enzymes that are involved in bacterial defense mechanisms [46]. Salicylic acid’s antimicrobial effects are thought to be related to its ability to interact with cell membrane proteins, disrupting chemiosmotic control and leading to cell death. It can also affect bacterial metabolism and potentially interfere with biofilm matrix formation [46]. Luteolin, a flavonoid, was found to exhibit both antimicrobial and antibiofilm properties by interfering with bacterial cell membranes, inhibiting biofilm formation, and potentially disrupting bacterial metabolism and energy production. It has been shown to be effective against various bacteria, including *Staphylococcus aureus* and *Listeria monocytogenes*, and can enhance antibiotic diffusion within biofilms [47]. Hypericin exhibits antimicrobial activity primarily through photodynamic inactivation and by inhibiting bacterial resistance mechanisms. It acts as a photosensitizer, generating reactive oxygen species (ROS) upon exposure to light, which damages and kills microbial cells. Additionally, hypericin can reduce the expression of genes involved in bacterial resistance, potentially enhancing the effectiveness of certain antibiotics [48]. Chrysin exerts antimicrobial activity affecting cell membrane integrity, inhibiting bacterial quorum sensing, and potentially enhancing the effectiveness of other antibiotics. It can also act as an antioxidant and anti-inflammatory agent, which can indirectly support antimicrobial efforts by reducing inflammation and oxidative stress [49]. 

As described above, several mechanisms support the herbal extract isolated compound’s ability to inhibit biofilm formation. Whole herbal extracts represent complex mixtures of compounds and could prove to have a similar or greater efficacy considering their potential to target multiple pathways associated with biofilm formation: bacterial adhesion, quorum sensing, and structural and functional components of the biofilm. Our present study employed a standardized qualitative protocol aimed at assessing the antibiofilm activity, thus further studies aimed at a multi-target approach and employing parameters such as subinhibitory concentrations (Sub-MICs) of extracts are intended to prove the mechanisms’ hypothesis. Such information is required to provide a comprehensive scientific base supporting *A. amica* antibiofilm potential applications.

## 4. Materials and Methods

### 4.1. Vegetal Material

The vegetal material was represented by the aerial parts and bulbs of *A. amica* (Pta and Ptb) from cultures located in Hoghilag village, Sibiu County, Romania (46°14′ N, 24°37′ E). The aerial parts were harvested during the flowering period (August 2022). During the yellowing of the leaves (autumn), the bulbs were dug up from the soil and cleaned. The vegetal products were dried in the shade in the laboratory. The species was identified at the Department of Pharmaceutical Botany of the “Iuliu Hațieganu” University of Medicine and Pharmacy in Cluj-Napoca, Romania, and was assigned voucher allocation number 202 [50,51,52,53].

### 4.2. Chemical Agents

Chemical reagents and solvents were purchased from several chemical companies. Thus, reagents aluminum chloride, Arnow and Folin–Ciocâlteu, ethanol, methanol, sodium acetate, hydrochloric and acetic acids, DPPH, acetonitrile, and ammonium acetate were purchased from MercK KGaA, Darmstadt (Germany). Ferric chloride III, hydroxide, and sodium carbonate were purchased from Alfa Aesar, USA, Germany, and 6-hydroxy-2,5,7,8-tetramethylchroman-2-carboxylic acid (Trolox) and 2,4,6-tripyridyl-s-triazine (TPTZ) from GmbH Steinheim (Steiheim, Germany). All references used in the LC–MS analysis were obtained from Phytolab (Vestenbergsgreuth, Germany). The spectrophotometer was Agilent Cary 60 UV–vis, (Santa Clara, CA, USA). Specific reagents for cytotoxicity and antimicrobial assays were obtained as follows: the DLD-1 cell line (CCL-221™) was purchased from the American Type Culture Collection, the RPMI-1640 medium was purchased from Gibco Life Technologies (Paisley, UK), fetal bovine serum and glutamine were purchased from Sigma-Aldrich (St. Louis, MO, USA), gentamicin and fluconazole were purchased from Oxoid Ltd., Hampshire, UK, the McFarland standard was purchased from Marcy l’Etoile (France), agar plates were purchased from Mueller–Hinton (MH), and Sabouraud dextrose was purchased from Darmstadt (Germany).

### 4.3. Extraction Method

The aerial parts (Pta) and bulbs (Ptb) were ground using a knife mill—Grindomix GM 200 (RETSCH GmbH, Haan, Germany) —and then extracted with 70% ethanol (70% ethanol in water *v*/*v*). The extracts were prepared hot, at 60 °C on a water bath, for 40 min, using 10 g of plant product and 100 mL of 70% ethanol. They were filtered into a volumetric flask and made up to 100 mL with 70% ethanol. Subsequently, the solutions were filtered through paper filters and centrifuged, and the supernatant solutions were collected and kept in the refrigerator until used for analysis [53,54,55,56]. Regarding the standardization of the extraction method, the extraction yield was calculated for each sample as follows: 27% for the Pta sample (g dry extract/g vegetal material), and for the Ptb sample, 40% (g dry extract/g vegetal material).

### 4.4. Total Polyphenolic Content (TPC) Quantification Method

The determination of the total polyphenolic content (TPC) of the two samples (Pta and Ptb) was spectrophotometrically performed, using the Folin–Ciocâlteu reagent in the presence of 29% sodium carbonate solution [52,54,57]. Absorbance was measured at 760 nm, and the results were calculated in mg gallic acid equivalents (GAE)/g dry plant material, using a gallic acid calibration curve (R^2^ = 0.999).

### 4.5. Flavonoid Content (FC) Quantification Method

The flavonoid content in the two *A. amica* extracts was evaluated by a spectrophotometric method using aluminum chloride (25%) and sodium acetate (10%), and the absorbance was measured at 430 nm. The final results were expressed in mg of rutin equivalents (RE)/g dry plant material, after establishing a calibration curve of the standard, rutin (R^2^ = 0.992) [52,54,58].

### 4.6. Caffeic Acid Derivatives (CADC) Quantification Method

To evaluate the content of caffeic acid derivatives in the two *A. amica* samples, the Arnow reagent (mixture of sodium nitrite and sodium molybdate) was used, the absorbance was measured at 500 nm, and the results were calculated using a caffeic acid calibration curve (R^2^ = 0.988). CADC values were expressed in mg caffeic acid equivalents (CAE)/g dry plant material [52,54,58].

### 4.7. Tannin Content (TC) Quantification Method

To evaluate the tannin content in the *A. amica* extracts, the indirect spectrophotometric method and the Folin–Ciocâlteu reagent were used, according to the European Pharmacopoeia, with some minor modifications [57]. First, the total polyphenol content (TPC) was determined. In the second step, after removing the insoluble tannin–protein complexes obtained with the hide powder reagent, the total content of non-tannin polyphenols (polyphenols not adsorbed on hide powder; TP non-TC) was determined. The tannin content (TC) was expressed as the difference between TPC and TP non-TC, in mg gallic acid equivalent (GAE)/g dry plant material [59,60].

### 4.8. LC–MS Analysis

The LC–MS analysis was performed using a Shimadzu Nexera I LC–MS-8045 (Kyoto, Japan) UHPLC system, equipped with a quaternary pump, an autosampler, an ESI probe, and a quadrupole rod mass spectrometer. Separation was carried out on a Luna C18 reversed-phase column (Phenomenex-Torrance, Torrance, CA, USA); the mobile phase consisted of a gradient of analytical-grade methanol, ultrapurified water, and formic acid. Detection was performed using a quadrupole rod mass spectrometer with electrospray ionization (ESI), both in negative and positive multiple reaction monitoring (MRM) ion modes. Identification was carried out via a comparison of the retention times, MS spectra, and the transitions between the separate compounds and references. Identification and quantification were based on the main transition from the MS spectra of each individual compound. Calibration curves (R^2^ = 0.9964–0.9999) were plotted for the quantification of compounds and references. The samples tested were injected in triplicate [51,53].

### 4.9. Antioxidant Assays

The estimation of the antioxidant potential of the *A. amica* extracts was established by two tests: the DPPH free radical method and the Ferric ion reducing antioxidant power (FRAP) assay.

#### 4.9.1. DPPH Test

The evaluation of the general antioxidant activity is based on the ability of the scavenger polyphenolic molecule to donate an electron to a stable organic radical of the purple DPPH• type, with its transformation into yellow DPPH_2_. The color change is spectrophotometrically measured at 517 nm using an ultraviolet–visible (UV/vis) spectrophotometer [61]. Method in brief: 2 mL of each extract (Pta and Ptb) of different concentrations were treated with 2 mL of methanolic DPPH solution (0.1 g/L). After half an hour, the absorbances of the controls (Ac) and samples (As) were determined at 517 nm. The percentages of inhibition of DPPH radicals were calculated, and the results were expressed as IC_50_ (mg/mL), i.e., the concentration of plant product that scavenges 50% of DPPH radicals [54].

#### 4.9.2. FRAP Test

The FRAP method developed by Benzie and Strain was adapted by Pulido R. et al. to measure the antioxidant capacity of plant extracts by reducing Fe3+ to Fe2+ in the presence of the 2,4,6-tripyridyl-s-triazine (TPTZ) radical [62,63]. The absorbance of the blue solution was measured at 450 nm. The results were expressed in μM Trolox equivalents/g dry plant material, using Trolox as a reference (R^2^ = 0.994) [54].

### 4.10. Cell Line and Cytotoxicity Assay

Colorectal adenocarcinoma cell line DLD-1 (CCL-221^TM^) was cultured in an RPMI-1640 medium (Gibco Life Technologies, Paisley, UK) supplemented with 10% fetal bovine serum (Sigma-Aldrich, St. Louis, MO, USA), 1% glutamine (Sigma-Aldrich, St. Louis, MO, USA), and 1% antibiotics-antimycotics (Gibco Life Technologies, Paisley, UK). Cells were incubated in a humidified atmosphere at 37 °C and 5% CO_2_. The antiproliferative potential of *A. amica* extracts (Pta, Ptb) was evaluated using the CCK-8 assay. DLD-1 cells (1 × 10^4^ cells/well) were treated with Pta and Ptb extracts at five distinct concentrations, expressed in μmol GAE/μL (considering the total polyphenol concentration, expressed in mg GAE/g). The concentrations used for Pta were calculated as 0.650 μmol GAE (25 μL extract—Pta1), 0.520 μmol GAE (20 μL extract—Pta2), 0.390 μmol GAE (15 μL extract—Pta3), 0.260 μmol GAE (10 μL—Pta4), and 0.130 μmol GAE (5 μL—Pta1), while for Ptb, the corresponding concentrations were 0.435 μmol GAE (25 μL extract—Ptb1), 0.348 μmol GAE (20 μL extract—Ptb2), 0.261 μmol GAE (15 μL extract—Ptb3), 0.174 μmol GAE (10 μL extract—Ptb4), and 0.087 μmol GAE (5 μL extract—Ptb5). The positive control consisted of doxorubicin (reference compound) at a concentration of 20 μg/mL, and the negative control was represented by cells cultured in a standard medium. In addition, the inhibitory effect of the solvent used for the preparation of the hydroalcoholic extracts was also assessed. After incubation for 24 h, CCK-8 solution (Sigma-Aldrich, St. Louis, MO, USA) was added to each well, and the cells were further incubated for 4 h at 37 °C in the dark. CCK-8, which contains a water-soluble tetrazolium salt, is reduced by viable cells to generate a colored formazan dye. The intensity of the chromogenic reaction correlates directly with the number of viable cells in the sample [64,65]. The absorbance of each well was subsequently measured at 450 nm using a microplate reader (Bio-Rad, Hercules, CA, USA). All experiments were conducted in triplicate and the results were expressed as the mean ± SD. The cell survival percentage was calculated based on the optical densities, with values compared to the optical density of the control group [54,66,67]. IC_50_ values were calculated by plotting cell viability against the logarithm of extract concentrations and fitting the data with a nonlinear sigmoidal dose-response curve using a four-parameter logistic model.

### 4.11. Antimicrobial Activity Assays

#### 4.11.1. Agar-Well Diffusion Method

The antimicrobial potential of *A. amica* extracts (Pta, Ptb) was initially evaluated based on the agar-well diffusion method [68] according to EUCAST (European Committee on Antimicrobial Susceptibility Testing) criteria [69]. The reference strains included were as follows: *Staphylococcus aureus* ATCC 25923 (methicillin-susceptible *S. aureus*, MSSA), *Staphylococcus aureus* ATCC 700699 (methicillin-resistant *S. aureus*, MRSA), *Bacillus cereus* ATCC 14579, *Enterococcus faecalis* ATCC 29219, *Escherichia coli* ATCC 25922, *Pseudomonas aeruginosa* ATCC 27853, and *Candida albicans* DSMZ 1386. The microbial reference strains were purchased from Oxoid Ltd. (Hampshire, UK). The assay included both positive and negative controls for the antimicrobial activity: gentamicin (10 µg) and fluconazole (25 µg) (Oxoid Ltd., Hampshire, UK) and 70% ethanol in water *v*/*v*, respectively. Each microbial strain was prepared as turbidity 0.5 McFarland (1.0 × 10^6^ CFU/mL) standard (bio-Meriuex, Marcy l’Etoile, France) equivalent inoculum and cultured onto suitable agars: Mueller–Hinton (MH) and Sabouraud dextrose (SD) (Merck, Darmstadt, Germany), for bacteria and *C. albicans*, respectively. Further, 6 mm diameter wells were cut into the inoculated agar plates to add in three wells for the volume of 50 μL for each tested product (extracts, negative control). Thus, considering the total polyphenol concentration, for this assay, the Pta and Ptb extract concentrations were calculated as 1.3 μmol GAE/μL and 0.870 μmol GAE/μL, respectively. After this step, the agar plates were incubated at 37 °C for 24 h for bacteria and 48 h for *C. albicans*, when the growth inhibition zone diameters were measured, and their corresponding values were recorded in mm. This *in vitro* test was performed in duplicate [70].

#### 4.11.2. Broth Microdilution Method

The *A. amica* extracts (Pta, Ptb) were further *in vitro* investigated using the broth microdilution method to determine the minimum inhibitory (MIC), bactericidal (MBC), and fungicidal (MFC) concentrations. This test involved preparing two-fold serial dilutions of each tested herbal extract using 100 µL of a specific broth (MH and SD, respectively) and sterile flat-bottomed 96-well microtiter plates (Deltalab, Barcelona, Spain). These dilutions were mixed with a volume of 5.0 µL microorganism inoculum to be incubated at 37 °C for 24 h for bacteria and 48 h for *C. albicans*; at the end of the incubation, the wells were visually examined against the controls (the two types of broths used to culture bacterial and fungal species (MH and SD, respectively)), considering the absence of turbidity indicative of *in vitro* inhibitory activity and recording its corresponding highest dilution as the MIC value. Further, a volume of 10.0 µL was sampled from each well, inoculated onto specific agar plates, and cultured for 24 h and 48 h for bacteria and *C. albicans*. MBC and MFC values were recorded for if the colonies were absent. Positive and negative controls were tested as well, namely gentamicin 50 mg/mL (Sigma-Aldrich, St. Louis, MO, USA), fluconazole (10–1000 μM) (Sigma-Aldrich, St. Louis, MO, USA), and 70% ethanol in water *v*/*v* with the evaluation made in duplicates for each tested product [70].

#### 4.11.3. Antibiofilm Assay

The *A. amica* extracts’ (Pta, Ptb) *in vitro* antimicrobial potential was also verified against the biofilm formation based on previous protocols [37,70,71]. Two stages, namely a biofilm attachment (T0) and a 24 h pre-formed biofilm (T24), were considered. In the case of T0, microbial inoculums were prepared using the same technique as described for the agar-well diffusion method [68] to be inoculated with equal volumes (100 μL) of each tested product in sterile flat-bottomed 96-well microtiter plates without shaking for 24 h at 37 °C. For T24, the plates were inoculated with each microbial inoculum and incubated for 24 h to obtain the formed biofilm stage that was further exposed to equal volumes of each extract. Similar to the other employed methods, this assay also included controls represented by organisms + specific broth, bacteria + MH broth + gentamicin, and *C. albicans* + SD broth + fluconazole. The antibiofilm activity for both stages was recorded based on the crystal violet staining (CVS) results. After 24 h incubation, for each well of the plate, the supernatant was removed, and the plates were washed three times using sterile distilled water. Following drying, the adhered cells were fixed with 96% methanol (150 μL) and further stained with a 0.1% crystal violet solution (100 μL) (Sigma-Aldrich, St. Louis, MO, USA). After 20 min at room temperature, these plates were washed with sterile distilled water and further treated with 150 μL of 100% ethanol. Finally, after gentle shaking, the optic density (OD) was recorded at 490 nm using a microplate reader Sunrise™ (Tecan, Männedorf, Switzerland), and the results were expressed as a percentage of inhibition based on the following equation: Inhibition (%) = (OD_control_ − OD_extract)_/OD_control_ × 100 [54]. These values (%) allowed the interpretation of the *in vitro* antibiofilm activity as good (above 50%, ++), poor (0–50%, +), and no inhibition or enhancement of biofilm development and growth (<0) [37].

### 4.12. Statistical Analysis

The described methods were performed in triplicate, and the results were presented as the mean standard using the Excel software package 2016. The results were subjected to one-way ANOVA for statistical analysis with the following values: *p* < 0.05, threshold value for statistical significance; *p* < 0.001, very significant; 0.001 < *p* < 0.05, significant; and *p* > 0.05, insignificant.

## 5. Conclusions

Our results showed important polyphenol content and caffeic acid as the main compound for tested *A. amica* ethanolic extracts. Significantly higher amounts of polyphenol compounds and also of antioxidant activity were found for the aerial parts extract compared to the bulbs one. Notable anti-*Candida albicans* activity and moderate efficacy against *Listeria monocytogenes* and *Staphylococcus aureus* were displayed by both extracts against planktonic cells and biofilm. A dose-dependent cytotoxicity towards the colorectal adenocarcinoma cell line was recorded as well. This study brings novelty to the scientific literature by characterizing the phenolic profile and the antimicrobial, antibiofilm, antioxidant, and antiproliferative activities of the ethanolic extracts obtained from *A. amica*, thus highlighting this herbal species’s medicinal potential.

## Figures and Tables

**Table 1:** Polyphenolic content and antioxidant activity of *A. amica* extracts.

*A. amica* Extracts	TPC (mg GAE/g)	TC (mg GAE/g)	FC (mg RE/g)	CADC (mg CAE/g)	DPPH (IC_50_ mg/mL)	FRAP (µM TE/g)
Pta	44.25 ± 1.08 ^e^	12.55 ± 0.34	9.20 ± 0.19	19.95 ± 0.05	0.82 ± 0.02 ^c^	79.75 ± 1.80
Ptb	29.60 ± 0.89 ^a,e^	9.85 ± 0.15 ^a^	5.65 ± 0.13 ^a^	8.08 ± 0.12 ^a^	1.65 ± 0.01 ^b,c^	29.62 ± 0.37 ^d^
Trolox	-	-	-	-	0.011 ± 0.002	-

Notes: Each value is the mean ± SD of three independent measurements; TPC: total polyphenolic content; TC: tannin content; FC: flavonoid content; CADC: caffeic acid derivatives content; GAE, RE, CAE, and TE: gallic acid, rutin, caffeic acid, and Trolox equivalents. Lowercase letters in the same row indicate significant differences: ^a^ *p* < 0.001 (Ptb vs. Pta); ^b^ *p* < 0.001 (Ptb vs. Pta); ^c^ *p* < 0.001 (Trolox vs. Ptb and Pta); ^d^ *p* < 0.001 (Ptb vs. Pta); ^e^ *p* < 0.001 (TPC vs. Ptb and Pta).

**Table 2:** Phenolic compounds identified by LC–MS analysis in the composition of the tested *A. amica* extracts (μg/g).

Compound	Retention Time, min		*m/z* and Main Transition		*A. amica* Content (μg/g)	
	Reference	Separated Compound	Reference	Separated Compound	Ptb	Pta
Caffeic acid	13.8	13.6	179.0 > 135.0	179.0 > 135.0	4230 ± 10.40	8890 ± 22.20
Chlorogenic acid	12.0	12.0	353.0 > 191.0	353.0 > 191.0	1501 ± 3.70	4000 ± 9.90
Salicylic acid	23.5	23.4	137.0 > 93.0	137.0 > 93.0	-	3400 ± 8.40
Carnosol	30.7	30.4	329.1 > 285.1	329.1 > 285.1	37 ± 0.20	34 ± 0.20
Chrysin	29.7	29.8	253.0 > 143.0	253.0 > 253.0	1380 ± 3.40	1920 ± 4.70
Hyperoside	20.3	20.4	463.1 > 300.0	463.1 > 300.0	1510 ± 3.70	1640 ± 4.10
Luteolin-*7-O*-glucoside	19.9	19.8	447.0 > 284.9	447.0 > 284.9	72 ± 0.40	2290 ± 5.70
Luteolin	26.9	26.8	287.0 > 153.0	287.0 > 153.0	-	2300 ± 5.70
Naringenin	26.3	26.1	271.0 > 119.0	271.0 > 119.0	-	45 ± 0.20
Quercetin	25.4	25.4	300.9 > 151.0	300.9 > 151.0	-	20 ± 0.09

Note: values represent the mean ± standard deviations of three independent measurements.

**Table 3:** *A. amica* extracts’ (Ptb, Pta) *in vitro* antimicrobial activity based on the agar-well diffusion method results.

Tested Samples	Diameters of Inhibition Zone (mm)							
	MSSA	MRSA	*Bacillus* *cereus*	*Enterococcus faecalis*	*Listeria monocytogenes*	*Escherichia coli*	*Pseudomonas* *aeruginosa*	*Candida albicans*
Ptb	13.25 ± 0.43 ^a^	10.75 ± 0.83 ^a^	12.75 ± 0.43 ^a^	0	14.25 ± 0.83 ^a^	10.5 ± 0.5 ^a^	0	19 ± 0.71 ^b^
Pta	13.50 ± 0.50 ^a^	11.50 ± 0.50 ^a^	11 ± 0.71 ^a^	0	14.45 ± 0.43 ^a^	10.25 ± 0.43 ^a^	0	18.5 ± 0.50 ^b^
Gentamicin	19 ± 0.00	17 ± 0.25	20 ± 0.00	10 ± 0.00	22 ± 0.50	19 ± 0.00	18 ± 0.00	-
Fluconazole	-	-	-	-	-	-	-	21 ± 0.00

Note: MSSA—methicillin-susceptible *Staphylococcus aureus*, and MRSA—methicillin-resistant *Staphylococcus aureus*. Values represent means of duplicate determinations (*n* = 2) ± standard deviations. Lowercase letters in the same column point out significant differences: ^a^ *p* < 0.05 (extract vs. gentamicin); ^b^ *p* > 0.05 (extract vs. fluconazole). Gentamicin (10 μg/disk) and fluconazole (25 µg/disk) were included as positive controls.

**Table 4:** *A. amica* extracts’ (Ptb, Pta) *in vitro* antimicrobial activity based on the broth microdilution assay.

Samples	Microorganisms															
	MSSA		MRSA		*Bacillus* *cereus*		*Enterococcus* *faecalis*		*Listeria* *monocytogenes*		*Escherichia* *coli*		*Pseudomonas* *aeruginosa*		*Candida* *albicans*	
	MIC	MBC	MIC	MBC	MIC	MBC	MIC	MBC	MIC	MBC	MIC	MBC	MIC	MBC	MIC	MFC
Ptb	1.3	2.6	1.3	2.6	1.3	2.6	>2.6	>2.6	1.3	1.3	2.6	> 2.6	>2.6	>2.6	0.325	0.325
Pta	1.3	2.6	1.3	2.6	2.6	2.6	2.6	>2.6	1.3	1.3	2.6	2.6	>2.6	>2.6	0.325	0.325
Gentamicin MIC (mg/L)	3		4		3		3		3		4		-		-	
Fluconazole MIC (mg/L)	-		-		-		-		-		-		-		8	

Note: “>D1” = not active at the tested concentration; MIC: minimum inhibitory concentration (μmol GAE/mL)/; MBC: minimum bactericidal concentration (μmol GAE/mL); MFC: minimum fungicidal concentration (μmol GAE/mL).

**Table 5:** *A. amica* extracts’ (Ptb, Pta) antibiofilm activity.

	Inhibitory Activity Against Biofilm					
Samples	MSSA		*Listeria monocytogenes*		*Candida albicans*	
	T0	T24	T0	T24	T0	T24
Ptb	+	++	+	++	++	++
Pta	+	++	+	++	++	++
Gentamicin	+	++	+	++	-	-
Fluconazole	-	-	-	-	++	++

Note: - = not active; + means poor and ++ means good antibiofilm activity.

## Data Availability

The original contributions presented in this study are included in the article/Supplementary Material; further inquiries can be directed to the corresponding author.

## Supplementary Materials

The following supporting information can be downloaded at: https://www.mdpi.com/article/10.3390/antibiotics14070638/s1, Figure S1: LC-MS chromatogram and mass fragmentation pattern obtained for the identification of caffeic acid in the Ptb sample; Figure S2. LC-MS chromatogram and mass fragmentation pattern obtained for the identification of chlorogenic acid in the Ptb sample; Figure S3. LC-MS chromatogram and mass fragmentation pattern obtained for the identification of carnosol in the Ptb sample; Figure S4. LC-MS chromatogram and mass fragmentation pattern obtained for the identification of chrysin in the Ptb sample; Figure S5. LC-MS chromatogram and mass fragmentation pattern obtained for the identification of hyperoside in the Ptb sample; Figure S6. LC-MS chromatogram and mass fragmentation pattern obtained for the identification of luteolin-7-O-glucoside in the Ptb sample; Figure S7. LC-MS chromatogram and mass fragmentation pattern obtained for the identification of caffeic acid in the Pta sample; Figure S8. LC-MS chromatogram and mass fragmentation pattern obtained for the identification of chlorogenic acid in the Pta sample; Figure S9. LC-MS chromatogram and mass fragmentation pattern obtained for the identification of salicylic acid in the Pta sample; Figure S10. LC-MS chromatogram and mass fragmentation pattern obtained for the identification of carnosol in the Pta sample; Figure S11. LC-MS chromatogram and mass fragmentation pattern obtained for the identification of chrysin in the Pta sample; Figure S12. LC-MS chromatogram and mass fragmentation pattern obtained for the identification of hyperoside in the Pta sample; Figure S13. LC-MS chromatogram and mass fragmentation pattern obtained for the identification of isoquercetin in the Pta sample; Figure S14. LC-MS chromatogram and mass fragmentation pattern obtained for the identification of luteolin-7-O-glucoside in the Pta sample; Figure S15. LC-MS chromatogram and mass fragmentation pattern obtained for the identification of naringenin in the Pta sample; Figure S16. LC-MS chromatogram and mass fragmentation pattern obtained for the identification of quercetin in the Pta sample.
