# Supplementary material for: Agave amica (Medik.) Thiede & Govaerts (Asparagaceae)—Insights into Its Valuable Phenolic Profile and In Vitro Antimicrobial, Antibiofilm, Antioxidative, and Antiproliferative Properties
Source: Antibiotics (Basel). 2025 Jun 23;14(7):638. doi: 10.3390/antibiotics14070638 (PMC12291647; doi:10.3390/antibiotics14070638)
Supplement: Supplementary file 1 [file antibiotics-14-00638-s001.zip › antibiotics-3679827-supplementary.pdf]

antibiotics

MDPI

Supplementary material

Agave amica (Medik.) Thiede & Govaerts (Asparagaceae) - Insights into its Valuable Phenolic Profile and *in vitro* Antimicrobial, Antibiofilm, Antioxidative and Antiproliferative Properties

Mihaela Niculae [1], Daniela Hanganu [2], * Ilioara Oniga [2], Sergiu-Alexandru Burcă [2], Brîndușa Tiperciuc [3], Irina Ielciu [4], Emoke Pall [1], Timea Bab [2,5], Ramona Flavia Burtescu [5], Mihaela Andreea Sava [6], Daniela Benedec [2]

[1] Department of Clinical Sciences, University of Agricultural Sciences and Veterinary Medicine Cluj-Napoca, 400372 Cluj-Napoca, Romania; mihaela.niculae@usamvcluj.ro (M.N.); emoke.pall@usamvcluj.ro (E.P.)
[2] Department of Pharmacognosy, Faculty of Pharmacy, “Iuliu Hattieganu” University of Medicine and Pharmacy, 400010 Cluj-Napoca, Romania; dhanganu@umfcluj.ro (D.H.); ioniga@umfcluj.ro (I.O.); burca.sergiu.alex@elearn.umfcluj.ro (S.-A.B.); bab.timea.henrietta@elearn.umfcluj.ro (T.B.); dbenedec@umfcluj.ro (D.B.)
[3] Department of Pharmaceutical Chemistry, “Iuliu Hațieganu” University of Medicine and Pharmacy, 41 V. Babeş Street, 400012 Cluj-Napoca, Romania; btiperciuc@umfcluj.ro (B.T.)
[4] Department of Pharmaceutical Botany, Faculty of Pharmacy, “Iuliu Hatieganu” University of Medicine and Pharmacy, 400337 Cluj-Napoca, Romania; irina.ielciu@umfcluj.ro (I.I.)
[5] PlantExtrakt Ltd., 407059 Cluj-Napoca, Romania; ramona.burtescu@plantextrakt.ro (R.F.B)
[6] Transylvania School of Botanic Art & Illustration, 557046 Copșa Mare, Romania; mihaelaandreea.sava@yahoo.com (M.A.S.)
* Correspondence: dhanganu@umfcluj.ro (D.H.)

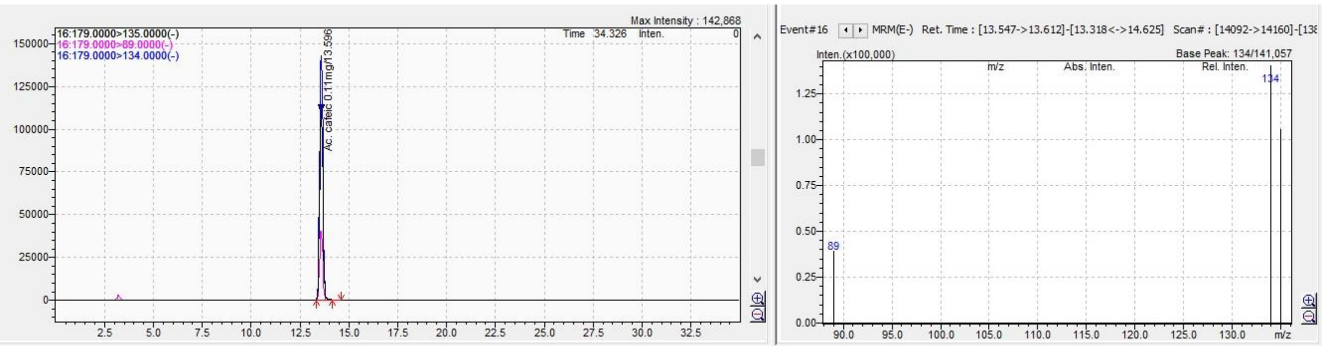

Figure S1. LC-MS chromatogram and mass fragmentation pattern obtained for the identification of caffeic acid in the Ptb sample

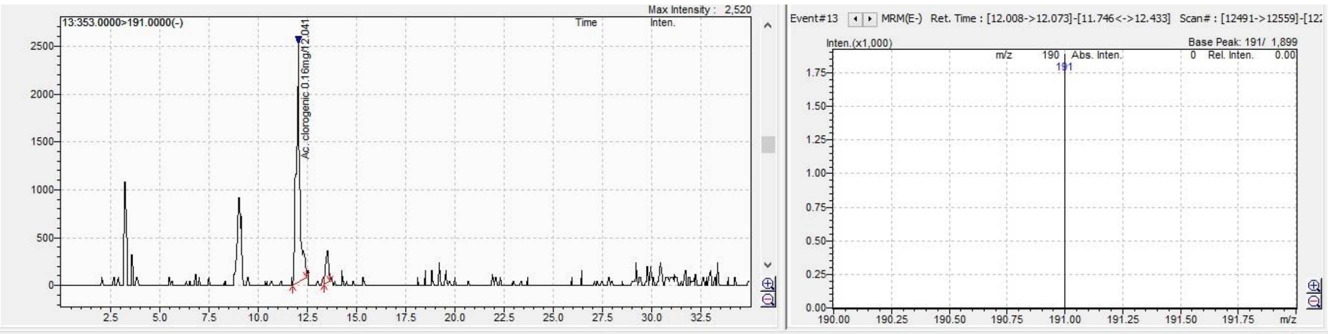

Figure S2. LC-MS chromatogram and mass fragmentation pattern obtained for the identification of chlorogenic acid in the Ptb sample

 https://doi.org/10.3390/xxxxx

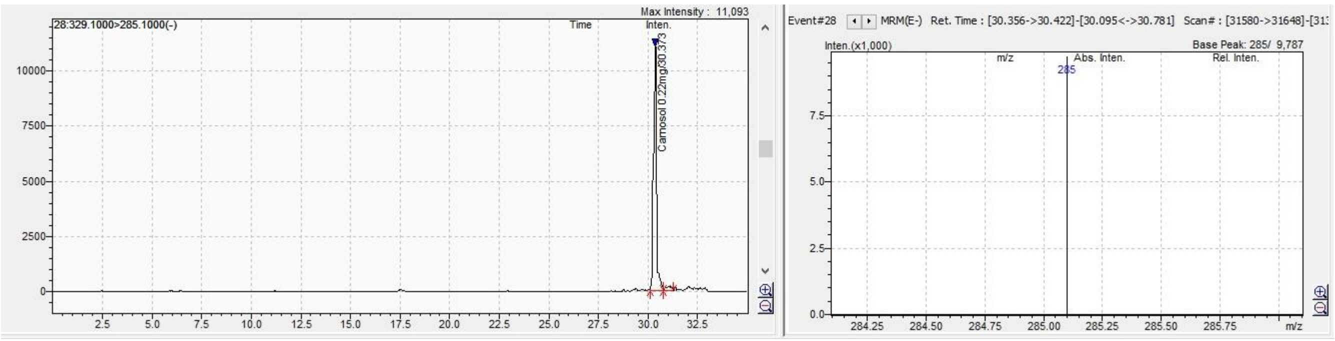

Figure S3. LC-MS chromatogram and mass fragmentation pattern obtained for the identification of carnosol in the Ptb sample

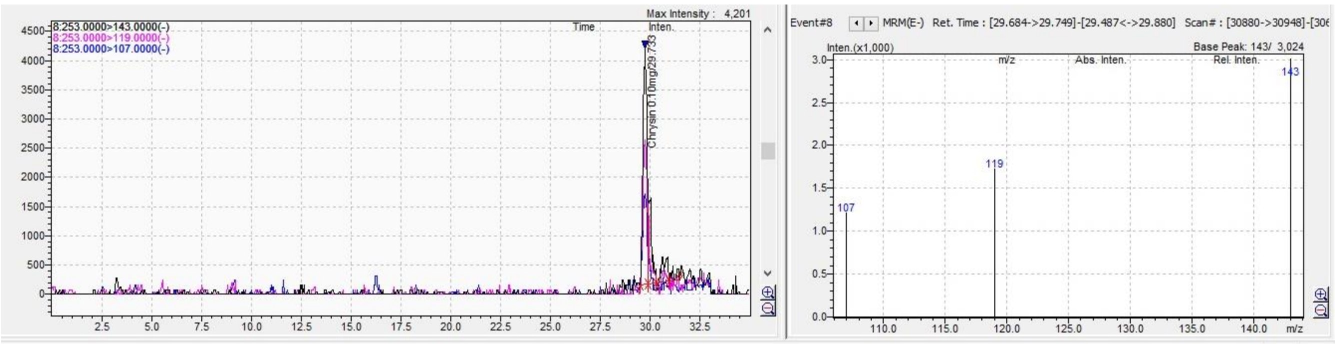

Figure S4. LC-MS chromatogram and mass fragmentation pattern obtained for the identification of chrysin in the Ptb sample

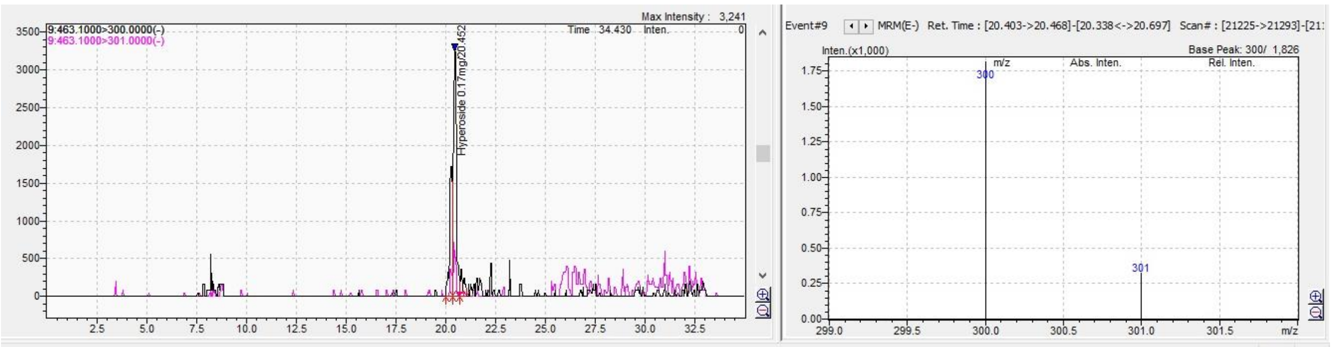

Figure S5. LC-MS chromatogram and mass fragmentation pattern obtained for the identification of hyperoside in the Ptb sample

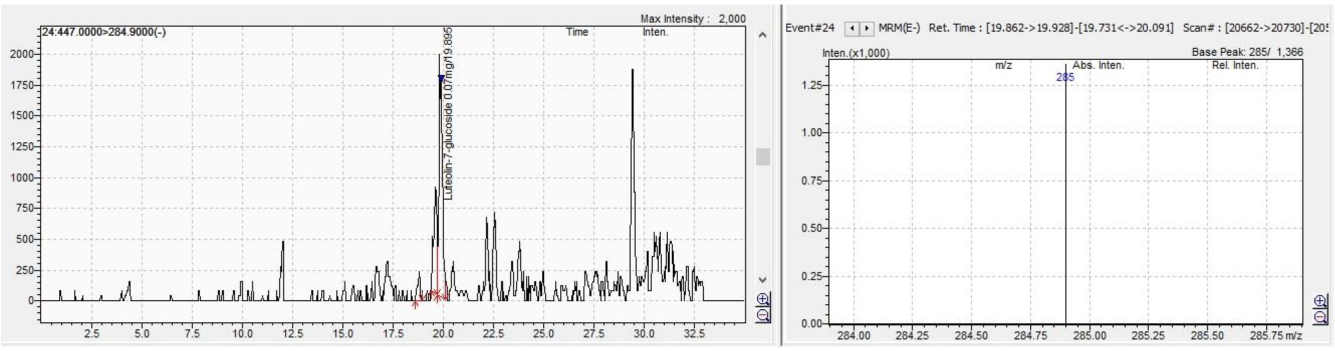

Figure S6. LC-MS chromatogram and mass fragmentation pattern obtained for the identification of luteolin-7-O-glucoside in the Ptb sample

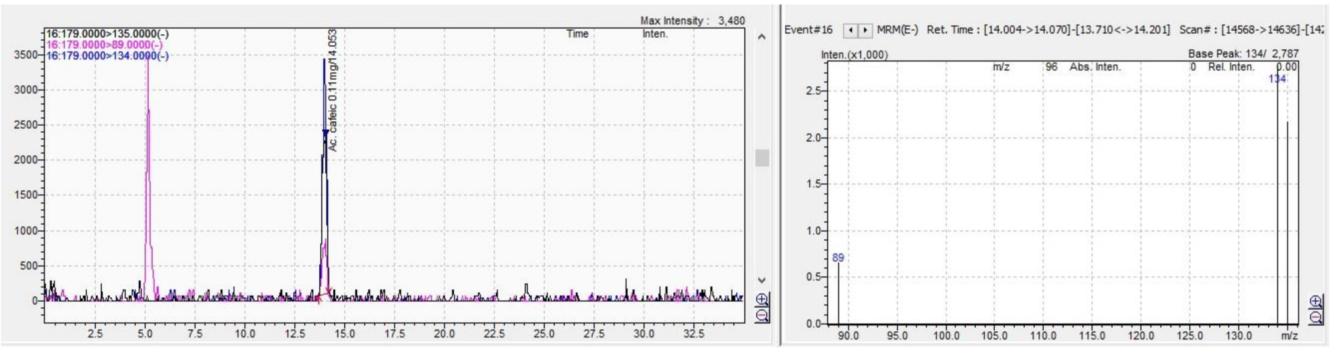

Figure S7. LC-MS chromatogram and mass fragmentation pattern obtained for the identification of caffeic acid in the Pta sample

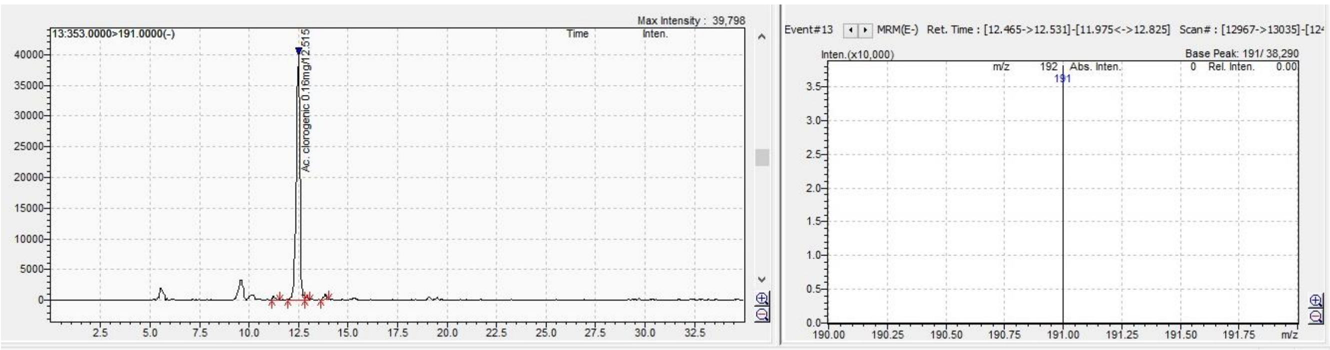

Figure S8. LC-MS chromatogram and mass fragmentation pattern obtained for the identification of chlorogenic acid in the Pta sample

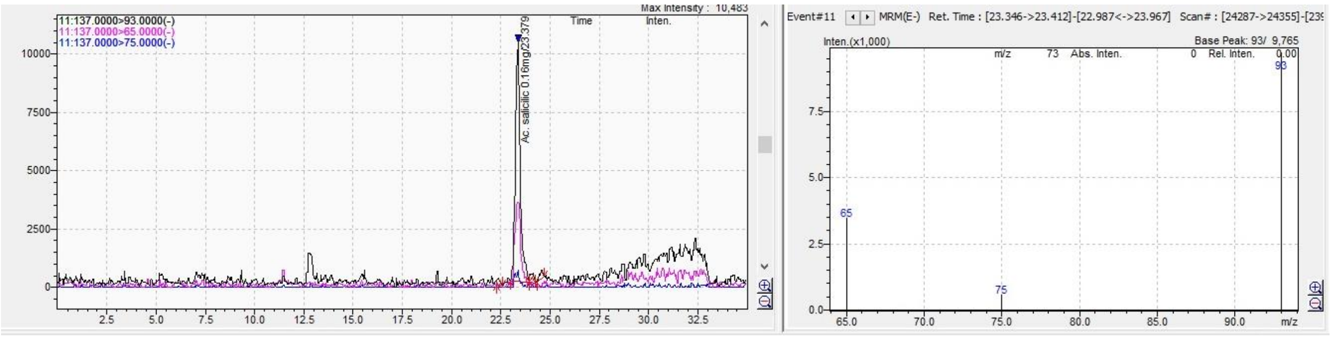

Figure S9. LC-MS chromatogram and mass fragmentation pattern obtained for the identification of salicylic acid in the Pta sample

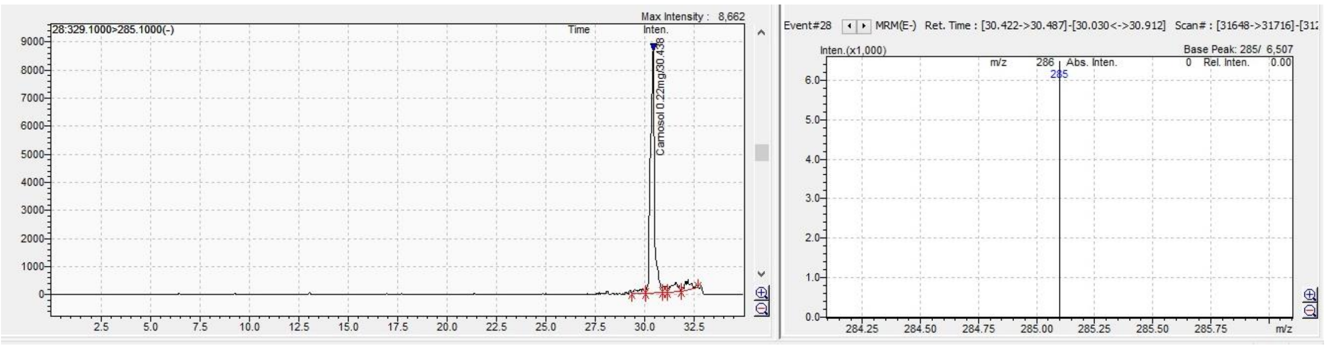

Figure S10. LC-MS chromatogram and mass fragmentation pattern obtained for the identification of carnosol in the Pta sample

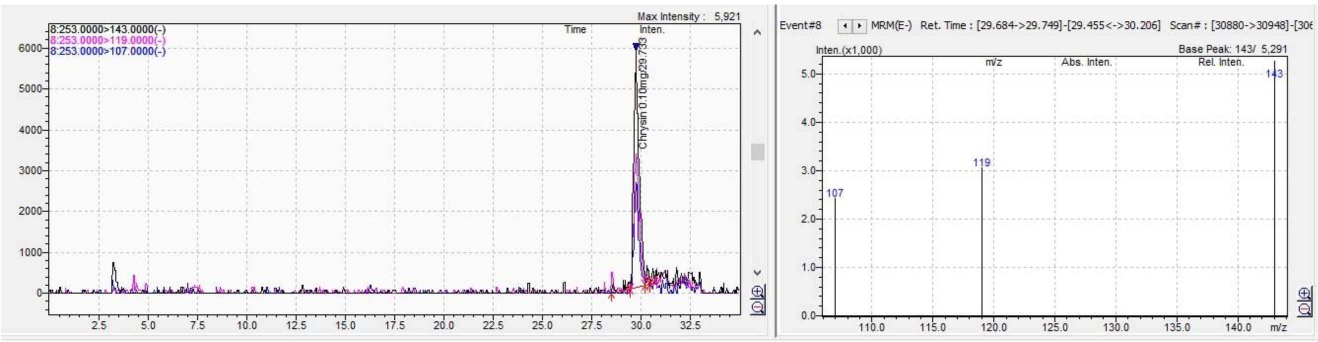

Figure S11. LC-MS chromatogram and mass fragmentation pattern obtained for the identification of chrysin in the Pta sample

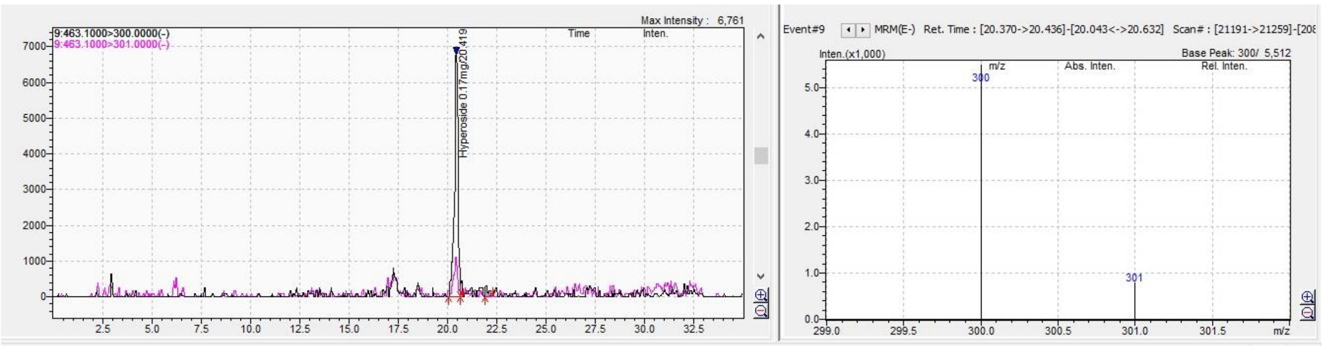

Figure S12. LC-MS chromatogram and mass fragmentation pattern obtained for the identification of hyperoside in the Pta sample

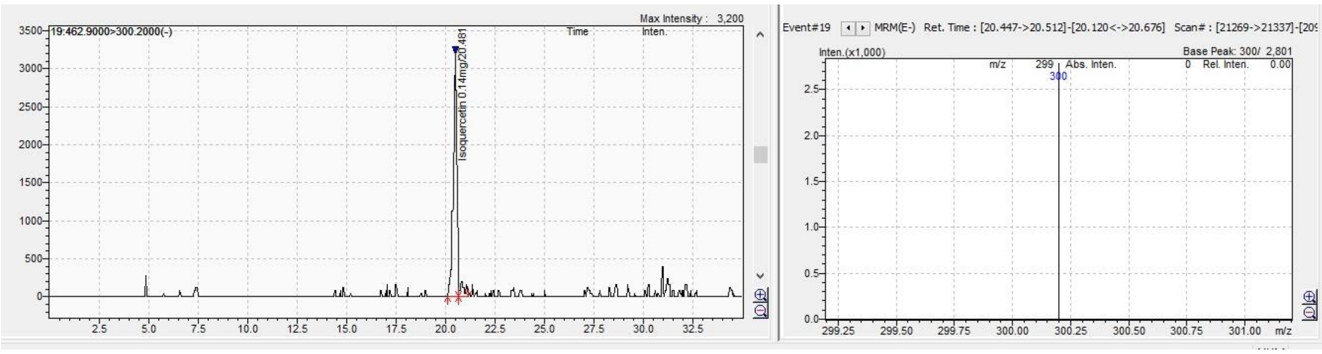

Figure S13. LC-MS chromatogram and mass fragmentation pattern obtained for the identification of isoquercetin in the Pta sample

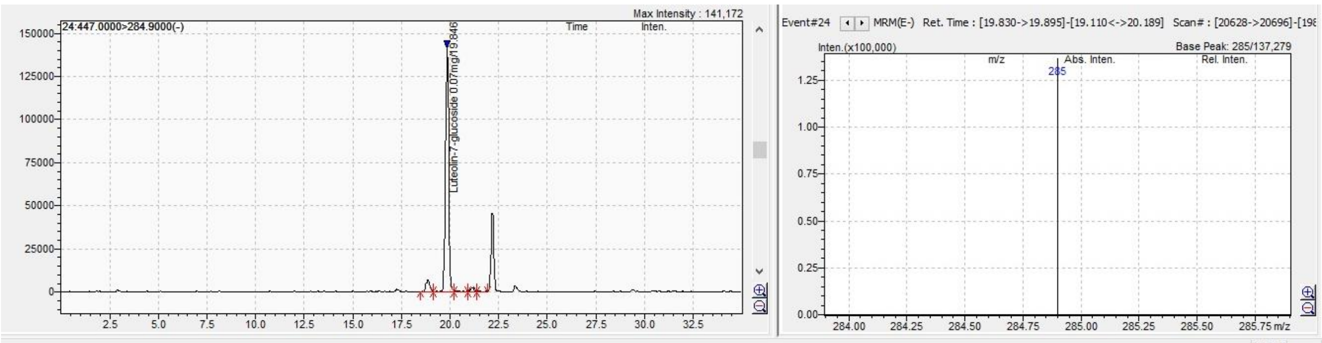

Figure S14. LC-MS chromatogram and mass fragmentation pattern obtained for the identification of luteolin-7-O-glucoside in the Pta sample

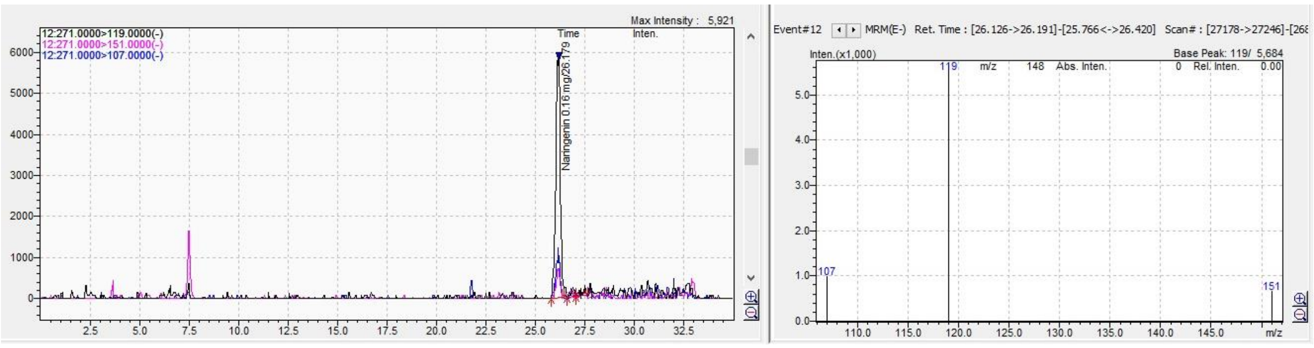

Figure S15. LC-MS chromatogram and mass fragmentation pattern obtained for the identification of naringenin in the Pta sample

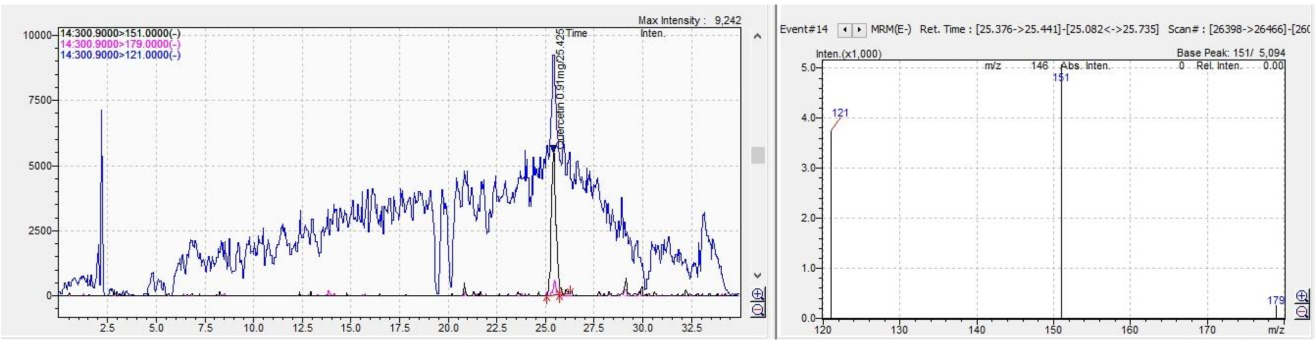

Figure S16. LC-MS chromatogram and mass fragmentation pattern obtained for the identification of quercetin in the Pta sample

Disclaimer/Publisher's Note: The statements, opinions and data contained in all publications are solely those of the individual author(s) and contributor(s) and not of MDPI and/or the editor(s). MDPI and/or the editor(s) disclaim responsibility for any injury to people or property resulting from any ideas, methods, instructions or products referred to in the content.
